# Comprehensive germline profiling of patients with breast cancer: initial experience from a Familial Cancer Clinic

**DOI:** 10.3332/ecancer.2024.1670

**Published:** 2024-02-15

**Authors:** Raja Pramanik, Sindhura Chitikela, S V S Deo, Ajay Gogia, Atul Batra, Akash Kumar, Ritu Gupta, Deepshi Thakral, Vedam L Ramprasad, Sandeep Mathur, D N Sharma, Aparna Sharma, Ashutosh Mishra, Babul Bansal

**Affiliations:** 1 Department of Medical Oncology, All India Institute of Medical Sciences, New Delhi 110029, India; 2 Department of Surgical Oncology, All India Institute of Medical Sciences, New Delhi 110029, India; 3 Department of Medical Oncology, National Cancer Institute, NCI Jhajjar 124105, India; 4 Department of Laboratory Oncology, All India Institute of Medical Sciences, New Delhi 110029, India; 5 Medgenome Labs Ltd, Bengaluru 560099, India; 6 Department of Pathology, All India Institute of Medical Sciences, New Delhi 110029, India; 7 Department of Radiation Oncology, All India Institute of Medical Sciences, New Delhi 110029, India; a https://orcid.org/0000-0003-0483-6254

**Keywords:** germline variant, next-generation sequencing, breast cancer, India

## Abstract

**Introduction:**

Breast cancer is the most common cancer among Indian females. There is limited data on germline profiling of breast cancer patients from India.

**Objective:**

The objective of the current study was to analyse the frequency and spectrum of germline variant profiles and clinicopathological characteristics of breast cancer patients referred to our Familial Cancer Clinic (FCC).

**Materials and methods:**

It is a single-centre audit of patients with a confirmed diagnosis of breast carcinoma referred to our FCC from January 2017 to 2020. All patients underwent pretest counselling. Genetic testing was done by multigene panel testing by next-generation sequencing along with reflex multiplication ligation-dependent probe amplification for BRCA1 and 2. The variants were classified based on American College of Medical Genetics guidelines. Demographic and clinicopathological details were extracted from the case record files.

**Results:**

One hundred and fifty-five patients were referred to the FCC and underwent pretest counselling. A total of 99 (63.9%) patients underwent genetic testing. Among them, 62 patients (62/99 = 62.6%) had a germline variant. A pathogenic/likely pathogenic (P/LP) germline variant was identified in 41 (41.4%) of the patients who underwent testing. Additional variants of unknown significance (VUS) were identified in seven patients who also carried a P/LP variant. VUS alone was detected in 21 patients (21/99 = 21.2%). Among the P/LP pathogenic variants (PV), BRCA 1 PV were seen in 27 patients (65.8%), BRCA 2 variants in 7 patients (17.1%), ATM variants in 3 patients (7.3%) and RAD51, TP53, CHEK2 and HMMR in 1 patient each. Variants were significantly more common in patients with a family history (FH) of malignancy than those without FH (58.5% versus 29.5%; *p* = 0.013). Age and triple-negative histology were not found to be significantly associated with the occurrence of P/LP PVs.

**Conclusion:**

We report a 41% P/LP variant rate in our selected cohort of breast cancer patients, with variants in BRCA constituting 83% and non-BRCA gene variants constituting 17%.

## Introduction

Breast cancer is the most common cancer representing 11.7% of cancers worldwide and the most common cause of cancer mortality in females [1]. It is the most common cancer among females in India [1, 2]. Genetic predisposition is the most important risk factor for the occurrence of breast cancer [3]. Approximately 10%–15% of all breast cancers can be attributed to the presence of germline pathogenic variants (PVs) involving BRCA and non-BRCA breast cancer susceptibility genes. Variants in BRCA 1 and 2 account for up to 50% of all familial breast cancer [4–6]. Other high-risk genes include PALB2, CDH1, PTEN, TP53 and STK11 with a lifetime risk of breast cancer of up to 50%–80%. Variants involving ATM, CHEK2, BRIP1, RAD51C, RAD51D, NBN and BARD1 are known to be associated with a moderately increased risk of breast cancer and a lifetime risk of around 25% [7, 8]. Testing and identification of these PVs is important to identify high-risk individuals, screening for early diagnosis of breast and/or ovarian cancer and accordingly plan for risk reduction and therapeutic strategies. There is limited literature on germline profiling in breast cancer cases, reported from India.

We report the results of a retrospective audit aimed at analysing the genetic and clinicopathologic profile of patients with breast cancer referred to a Familial Cancer Clinic (FCC) at a tertiary care cancer centre in India.

## Methods

This retrospective observational study was carried out at a tertiary care centre in India. Patients with histopathological diagnosis of carcinoma breast who were referred to our genetic clinic between January 2017 and 2020 were analysed in the study. Pre-test counselling and post-test counselling were done by a team of medical oncologists and surgical oncologists. The genetic test performed included multigene panel testing by next-generation sequencing (NGS) along with reflex multiplication ligation-dependent probe amplification (MLPA) to detect large deletions/duplications in BRCA1 and 2. Those with pathogenic/likely pathogenic (P/LP) variants in cancer predisposition genes were offered post-test counselling and further management was formulated in the multidisciplinary tumour board. The variants were classified based on American College of Medical Genetics (ACMG) guidelines.

### Genetic testing

Briefly, blood samples (8 mL) were collected from each patient and DNA extraction was done. A targeted gene panel of 104 genes (listed in Supplementary Table S1) was performed using a custom capture kit. Gene libraries were sequenced to mean > 80–100× coverage on the Illumina sequencing platform. Sequences were aligned to the human reference genome (GRCh38.p13). Gene annotation of the variants was performed using Ensembl Variant Effect Predictor programme against the Ensembl release 99 human gene model. Reflex MLPA testing was done for patients with no identifiable germline variants. Copy number variations in 24 exons of BRCA 1 and 27 exons of BRCA 2 were identified by hybridising with MLPA assay. Each MLPA probe consisted of two hemi-probes bound to an adjoining site of the target sequence. Upon ligation and PCR amplification, an amplicon of a unique length was generated by each probe. Copy number changes of different exons between test and control DNA samples were analysed by detecting the MLPA peak pattern. Variants identified were classified as pathogenic (class 5), likely pathogenic (class 4), variants of unknown significance/VUS (class 3), likely benign (class 2) and benign (class 1) according to ACMG guidelines. All P/LP variants were reported using the Human Genome Variant Society (HGVS) nomenclature and the most frequent transcripts. For example, NM_7297.4 (7,088 bp) and NP_009225.1 (1,863 amino acids) for BRCA1 germline variants.

Data regarding demographic characteristics, clinical profile, histopathology and family history (FH) were extracted from the files in the medical record system.

The primary outcome was the frequency of P/LP PVs in the cohort. Secondary outcomes included the identification of clinicopathological features, their association and the spectrum of PVs.

### Statistics

Categorical data were represented using descriptive statistics in the form of percentages, medians and ranges. Numerical data were represented as median and range. Chi-square tests and Fisher’s exact tests were used to compare and examine the relationship between variables. All tests of the hypothesis were conducted at an alpha level of 0.05, with a 95% confidence interval. Statistical analysis was performed using IBM SPSS version 26 software.

## Results

A total of 155 patients were referred to our genetic clinic during this period. Most of the patients (*n* = 151) were referred from the breast cancer clinic of AIIMS New Delhi or NCI-AIIMS, Jhajjar. Four patients were referred from the oncology clinics of Army Hospital (R&R) in New Delhi. The median age of the entire cohort was 42 years (range 21–80 years). All patients were female except one.

Pre-test counselling was done for all the patients and all 155 qualified the contemporary National Comprehensive Cancer Network (NCCN) criteria for genetic testing. All 155 patients were advised genetic testing by a multigene NGS panel. Ethnicity was assessed by their religion and place of residence. There were 129 (83.22%) Hindus, 8 (5.16%) Muslims, 6 (3.87%) Christians and 11 (7.09%) Sikhs. Overall, 59% of patients were from urban areas and 58% were literate.

Eight patients had bilateral breast cancer with synchronous breast cancer in three of them, while seven patients had both breast and ovarian cancer (Table 1).

Information on FH was available for 107 patients. A FH of breast and/or ovarian cancer in first-degree relatives was present in 35 patients (32.71%). A FH of malignancies other than breast and ovarian cancer was seen in 11 patients (10.28%). The FH of malignancy in second-degree relatives was present in a total of 42 patients (39.25%) (Table 1).

A total of 99 (63.9%) patients underwent genetic testing. The reasons for not undergoing testing included financial issues (50/56 = 89%) and patient preference (6/50 = 11%). Of these 99, 62 patients (62/99 = 62.6%) had a germline variant. As a whole, 73 variants were identified in 62 patients. Of those with variants, most of the patients (52/62 (87%)) had a single variant, with two variants in nine (14.5%) and three variants in one patient (1.6%), respectively (Table 2).

A P/LP variant was identified in 41 (41/99 = 41.4%) of the patients who underwent testing. Additional VUS were identified in seven patients who also carried a P/LP variant. VUS alone were detected in 21 patients (21/99 = 21.2%). Two patients had VUS involving 2 genes and 1 patient had a VUS each in 3 genes accounting for a total VUS variants of 25 (Table 2 and Supplementary Table S2).

### Clinical characteristics of patients with P/LP variants (n = 41)

The median age of patients with P/LP variants was 42 years. Nineteen patients (46.34%) belonged to the age group of less than or equal to 40 years. Information about FH was available for 31 patients amongst whom a FH of breast and/or ovarian cancer was observed in 24 patients (77.4%). Receptor status was available for 37 patients; amongst whom, triple-negative breast cancer (TNBC) was identified in 26 patients (70.3%), hormone-positive breast cancer in 9 patients (24.3%) and triple-positive breast cancer in 2 patients (5.4%) (Table 1, Supplementary Figure S2-S4).

**Figure 1.**
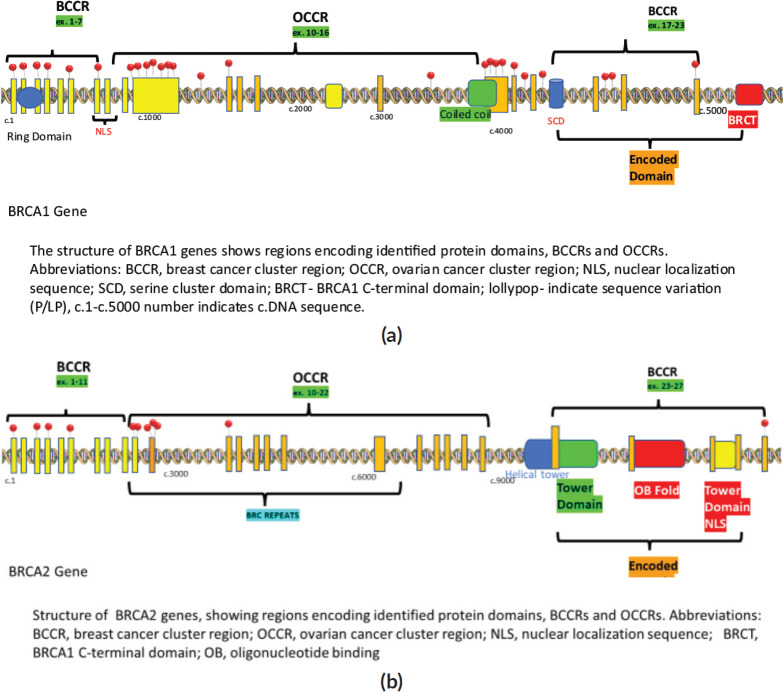
Lollipop plot of the detected P/LP variants during the study. (a): BRCA1 gene. (b): BRCA2 gene.

**Supplementary Figure S1.**
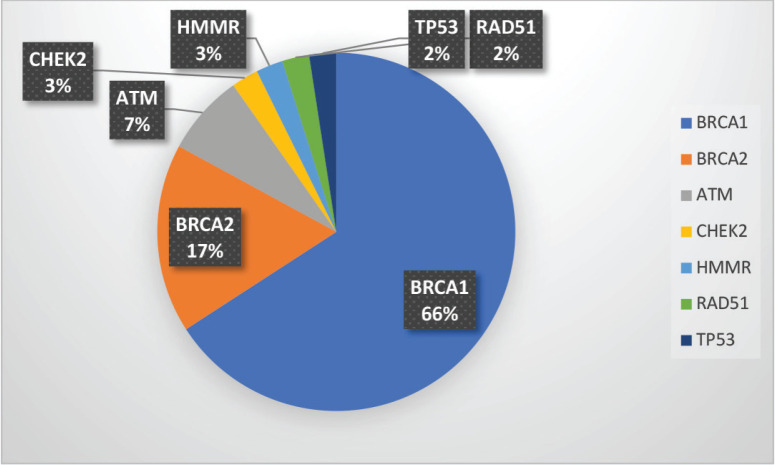
Spectrum of P/LP mutations

**Supplementary Figure S2.**
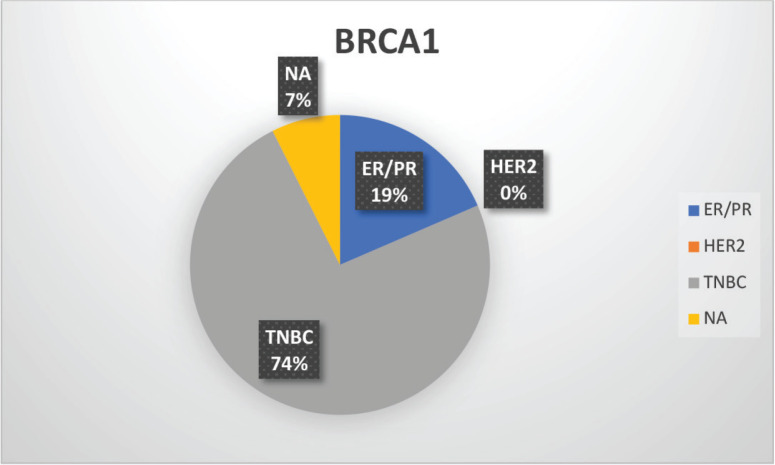
Distribution of subtypes in BRCA1 patients.

**Supplementary Figure S3.**
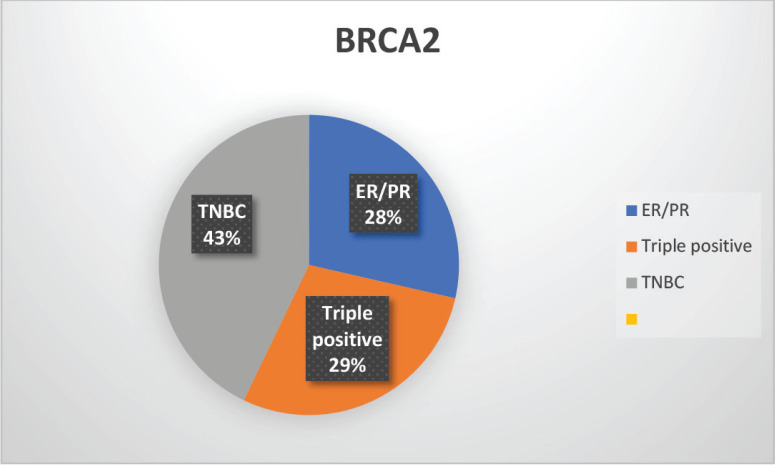
Distribution of subtypes in BRCA2 patients.

**Supplementary Figure S4.**
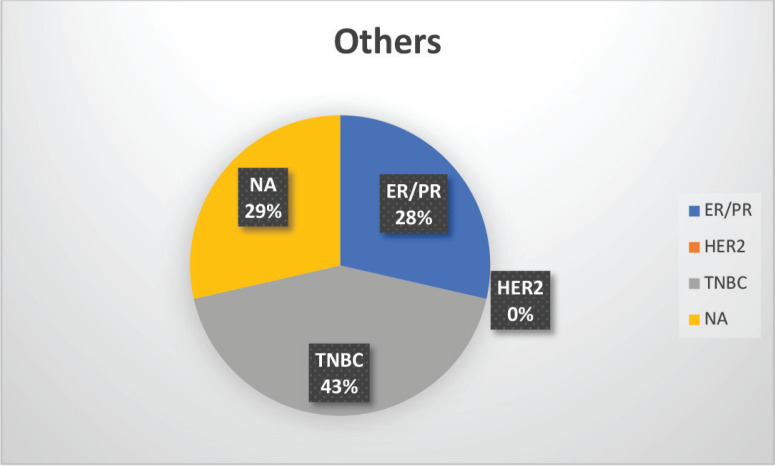
Distribution of subtypes in non-BRCA mutation patients.

Among the P/LP variants, BRCA 1 variants were seen in 27 patients (65.8%), BRCA 2 variants in 7 patients (17.1%), ATM variants in 3 patients (7.3%) and RAD51, TP53, CHEK and HMMR in 1 patient each (Table 2, Supplementary Figure S1). The Ashkenazi Jews founder variant (c.68_69delAG) was seen in two patients. Large genomic rearrangement in BRCA1 were found in two patients (Supplementary Table S3, Figure 1).

### Association of P/LP variants with FH, histology and age at diagnosis

Out of the 41 patients with a positive FH who underwent genetic testing, 24 patients had a P/LP variant (58.5%) compared to 7 patients among 27 patients who had no positive FH and underwent genetic testing (25.9%). Variants were significantly more common in patients with a FH of malignancy (*p* = 0.013) (Table 3).

P/LP variants were present in 26 out of 57 tested patients with TNBC (45.6%). This was not significantly different from that of the non-TNBC cluster, in which P/LP variants were present in 11 out of 31 tested patients (35.48%) (*p* = 0.358) (Table 3). P/LP were present in 19 out of 48 patients tested in the age group ≤40 years (42.68%) and in 22 out of 51 patients tested in the age group >40 years (40%). The difference was not significant (*p* = 0.721) (Table 3).

### Hereditary breast-ovarian cancer syndrome

Out of the seven patients who had both breast and ovarian cancer (metachronous), five had undergone genetic testing, with P/LP variant found in all five patients (four BRCA1 and 1 BRCA2). Out of the eight patients who had bilateral breast cancer, either synchronous or metachronous, six underwent genetic testing, out of which four had P/LP variants (three BRCA1, 1 TP53).

## Discussion

The current study is an audit of patients with breast cancers who were selectively referred to our FCC over the initial 3 years of its functioning.

We report 41% positivity for P/LP PVs in this highly selected cohort of patients with carcinoma breast. BRCA 1 constituted the most mutated gene and accounted for 65%, followed by BRCA2 variants in 13.7% and non-BRCA genes constituted 22.6%. The prevalence of the Ashkenazi Jew founder variant was found to be 7%.

In a retrospective study by Singh *et al* [9], around 1,010 patients with breast and ovarian cancers were tested using a 14-gene panel, germline variants were identified in 30% of patients, with variants in BRCA genes constituting 84.9% and that of non-BRCA genes constituting 15.1%. They reported significantly higher variant detection rates in the age group of 40 years or younger, TNBC and in those with first-degree family members affected with breast and/or ovarian cancer [9]. Another study by Chheda *et al* [10] is a laboratory-based multicentre study of 160 unrelated women with breast and ovarian cancer or unaffected women with a FH. Variant testing was done for only BRCA 1 and BRCA 2 and they reported a frequency of 31.9% for P/LP PVs. A similar study from a tertiary care centre reported a comparable variant rate involving BRCA 1 in 22% and BRCA 2 in 8% [11]. Both the above-cited studies showed comparable variant detection rates to our study. However, there is a higher percentage of non-BRCA variants reported in our study 22.6% versus 15% in the study by Singh *et al* [9] and 11.9% in a study by Kadri *et al* [12] which could be attributed to the application of an extended targeted gene panel in our study. In a prospective study by Mittal *et al* [13], conducted at a tertiary centre in North India in which consecutive carcinoma breast patients (*n* = 236) were subjected to multigene hereditary germline panel testing, P/LP variants were found in 44/236 (18.64%) women; variants in BRCA1 (22/47, 46.8%) and BRCA2 (9/47, 19.1%) were the most common, with 34% of variants present in non-BRCA genes. This prevalence of 18.64% in unselected breast carcinoma patients may actually be close to the true prevalence of germline variants in the population. The variant detection rate is higher in our study because of the highly selected study population.

The prevalence of germline variants is significantly higher compared to that of reports from various ethnicities worldwide. A study by Peto *et al* [5] reported a prevalence of BRCA 1 and BRCA 2 of 3% in unselected breast cancer patients, respectively, in the UK with higher prevalence in those aged <36 years. A study by Tung *et al* [14] reported that among sequential patients with breast cancer (*n* = 488) from the USA, germline variants in cancer predisposition genes were found in 10.7%, using a panel of 25 predisposition genes including 6.1% in BRCA1/2 and 4.6% in other breast/ovarian cancer predisposition genes including CHEK2 (*n* = 10), ATM (*n* = 4), BRIP1 (*n* = 4) and one each in PALB2, PTEN, NBN, RAD51C, RAD51D, MSH6 and PMS2. BRCA variant rate was higher in patients of young age, of Ashkenazi Jewish ancestry, with TNBC and those with a FH of breast/ovarian cancer [14]**.** Another German study by Engel et al [15] on the prevalence of germline BRCA 1/2 variants in TNBC patients with no FH reported a variant prevalence of 15.8% in the overall cohort, 32.9% in the age group 20–29 years, significantly higher compared to 6.9% in the age group 60–69 years. In a Chinese study reported by Sun *et al* [6], 8,085 consecutive unselected breast cancer patients were enrolled, and germline variants were assessed using a 62-gene panel. P/LP PVs were identified in 9.2% of patients, 5.3% of patients had *BRCA1* or *BRCA2* variant (1.8% in *BRCA1* and 3.5% in *BRCA2*) and 3.9% carried variants in non-BRCA genes with the highest prevalence in TNBCs. In a systematic review by Armstrong *et al* [16] of 70 studies, BRCA1/2 variant prevalence was reported to be around 1.8% in Spain to 15.4% in the United States, with a higher prevalence in TNBCs.

Our study is a retrospective analysis with a small sample of a highly selected group of patients preferentially referred for testing. The present study shows a high prevalence of variants in breast cancer predisposition genes in our setting and represents an audit of our initial experience of the FCC. The non-significant difference in the frequency of variants based on age and hormone status contrasts with the findings reported from other Indian studies as well as studies from other regions of the world [6, 9, 14, 15]. This may be attributed to the high selection and referral bias in our patient population.

With the advent of targeted therapies like poly (ADP-ribose) polymerase inhibitors in early as well as metastatic breast cancers, germline testing for breast cancer patients plays a pivotal role in the management of breast cancer [17–20]. NCCN [21] criteria for genetic testing have been substantially relaxed to the extent that almost every breast cancer patient merits a germline multi-gene panel testing.

The evidence quoted above may indicate a higher prevalence of germline variants in our setting compared to Western cohorts. Larger data using multi-gene panels in an unselected manner is the need of the hour to better qualify the exact prevalence and spectrum.

Variants in the South Asian population particularly India are less well represented in Western studies making variant calling difficult.

## Conclusion

This initial experience from a newly established FCC at a tertiary cancer centre can serve as an example to understand practical and logistic real-world challenges in gathering data as well as help us in planning larger multicentre studies. Such studies are extremely relevant to assimilate India-specific data and knowledge about various variant and their clustering if any in the Indian subcontinent. Also, smaller sample sizes and referrals from clinics highlight the unmet need for clinician awareness in proactively pursuing genetic evaluations in breast cancer patients. Mainstreaming genetic testing into routine clinical care is the way forward to cater to our huge clinical burden.

## Conflicts of interest

All the authors declare no potential conflict of interest.

## Funding

Not applicable.

## Ethical approval

The study protocol was approved by the Institute Ethics Committee vide letter number: IEC- 511/05.06.2020, RP-50/2020 dated 30.9.2020.

## Consent to participate

Informed consent was obtained from all patients.

## Author contributions

Authors, RP, SVSD, AG, AB, AK, AS, RG, DT, SM and VR contributed to the concept design and conduct of the study; RP, SC, BB, AM and AS did genetic counselling; and SC, AS and RP did the statistical analysis. SC and RP drafted the manuscript and all authors edited the manuscript.

## Availability of data and material

Data regarding this study will be available from the corresponding author (RP) on reasonable request.

## Figures and Tables

**Table 1.** Baseline characteristics.

Baseline characteristics (*n* = 155)		Median/mean ± SD/percentage
Age		42 years (range 28–80)
Gender		
	Female	154 (99.35)
	Male	1 (0.61)
Ethnicity		
	Hindu	129 (83.22)
	Muslim	8 (5.16)
	Christian	7 (3.87)
	Sikh	11 (7.09)
	Jain	1 (0.64)
Marital status		
	Married	140 (90.32)
	Unmarried	15 (9.68)
Area of residence		
	Rural	64 (38.78)
	Urban	97 (58.78)
	Not known	4 (2.4)
Hormone receptor status		
	Non-TNBC	57 (36.8)
	* Her2 Neu positive*	* 6*
	* ER/PR positive*	* 43*
	* Triple positive*	* 8*
	TNBC	78 (50.3)
	Not available	20 (12.9)
Test advised		
	Done	99 (63.90)
	Not done	56 (36.10)
Variants (*N* = 99)		
	Present	62 (62.6)
	Absent	37 (37.4)
Number of variants (73)		
	One mutation	52 (87)
	Two mutations	9 (14.5)
	Three mutations	1 (1.6)
Bilateral breast cancer		8
Synchronous bilateral breast cancer		3
Ovarian cancer (any time)		7
FH in FDR (number affected) (*N* = 107)		
Breast cancer		
	1	25
	2	3
	3	1
Ovarian cancer		
	1	10
	2	1
Other		
	1	17
	2	3
Surgical risk reduction		
	RRM advised	8
	RRM done	3
	RRSO advised	10
	RRSO done	6

**Table 2.** Spectrum of variants.

P/LP		
	P/LP	34
	VUS	21
	Both P/LP and VUS	7
Locus of P/LP variants (*N* = 41)		
	BRCA1	27 (66%)
	BRCA2	7 (17%)
	ATM	3 (7%)
	CHEK	1 (2.4%)
	RAD51	1 (2.4%)
	HMMR	1 (2.4%)
	TP53	1 (2.4%)
VUS (21 patients, 25 variants)		
	ATM	2 (9.5%)
	BRCA1	1 (4.8%)
	BRCA2	3 (14.3%)
	CDH1	2 (9.5%)
	CHEK2	1 (4.8%)
	ERCC2	1 (4.8%)
	FANCA	1 (4.8%)
	FANCE	1 (4.8%)
	FANCI	1 (4.8%)
	MLH1	1 (4.8%)
	MLH3	1 (4.8%)
	HMMR	1 (4.8%)
	MRE	1 (4.8%)
	MSH6	2 (9.5%)
	NBN	1 (4.8%)
	PALB2	1 (4.8%)
	RAD50	3 (14.3%)
	RAD51	1 (4.8%)
	RAD51C	1 (4.8%)

**Table 3.** Association of P/LP mutation with age, histology and FH.

Patient characteristics	Positive for P/LP variant	Negative for P/LP variant	*p*-value
Age (n = 99)			0.721
Age ≤ 40 years	19/48 (39.6%)	29/48 (60.4%)	
Age > 40 years	22/51 (43.1%)	29/51 (56.9%)	
Histology (n = 88)			0.358
TNBC	26/57 (45.6%)	31/57 (54.4%)	
Non-TNBC	11/31 (35.5%)	20/31 (64.5%)	
FH (n = 68)			0.013
Positive FH	24/41 (58.5%)	17/41 (41.5%)	
No FH	7/27 (25.9%)	20/27 (74.1%)	

**Supplementary Table S1.** List of genes in the targeted germline panel used during the study.

AIP	ALK	APC	AR	ATM	BAP1	BARD1	BLM	BMPR1A	BRCA1
BRCA2	BRIP1	BUB1B	CD82	CDC73	CDH1	CDK4	CDKN1C	CDKN2A	CEBPA
CEP57	CHEK2	CYLD	DDB2	DICER1	DIS3L2	EGFR	ELAC2	ENG	EPCAM
ERCC2	ERCC3	ERCC4	ERCC5	EXT1	EXT2	EZH2	FANCA	FANCB	FANCC
FANCD2	FANCE	FANCF	FANCG	FANCI	FANCL	FANCM	FH	FLCN	GATA2
GPC3	HRAS	KIF1B	KIT	MAX	MEN1	MET	MLH1	MLH3	MSH2
MSH3	MSH6	MUTYH	NBN	NF1	NF2	NSD1	PALB2	PHOX2B	PMS1
PMS2	PRF1	PRKAR1A	PTCH1	PTEN	RAD50	RAD51C	RAD51D	RB1	RECQL4
RET	RHBDF2	RNASEL	RUNX1	SBDS	SDHA	SDHAF2	SDHB	SDHC	SDHD
SLX4	SMAD4	SMARCB1	STK11	SUFU	TMEM127	TP53	TSC1	TSC2	VHL
WRN	WT1	XPA	XPC						

**Supplementary Table S2.** Patient-wise spectrum of VUS mutations.

S. no.	Age	P/LP	Exon	Loci of P/LP	Protein
1	40	NBN FANCI	Exon 5 Exon 36	c.562A>T c.3785_3789del	p.Lys188Ter p.Lys1262ThrfsTer6
2	30	BRCA2	Exon 6	c.487A>C	p.Ser163Arg
3	50	CDH1	Exon 3	c.304G>A	p.Ala102Thr
4	41	BRCA2	Exon 12	c.6935A>T	p.Asp2312Val
5	32	MLH3 CDH1	Exon 2 Exon 3	c.1870G>C c.304G>A	p.Glu624Gln p.Ala102Thr
6	80	ATM	Exon 13	c.1975A>G	p.Lys659Glu
7	45	FANCA	Exon 42	c.4168G>G/C	p.Gly1390Arg
8	44	FANCE	Exon 2	c.350_351del	p.Val117AlafsTer11
9	61	HMMR	Exon 10	c.983T>A	p.Leu328His
10	55	ATM	Exon 29	c.4303A>C	p.Lys1435Gln
11	40	CDH1	Exon 9	c.1223C>T	p.Ala408Val
12	30	RAD50	Exon 19	c.3011T>C	p.Met1004Thr
13	45	MLH1	Exon 13	c.1420C>T	p.Arg474Trp
14	36	BRCA1	Exon 11	c.4105G>A	p.Ala1369Thr
15	34	NBN	Exon 10	c.1147G>A	p.Glu383Lys
16	35	RAD51C	Exon 1	c.52C>T	p.Pro18Ser
17	40	BRCA2	Exon 27	c.10082A>C	p.Gln3361Pro
18	39	FANC1	Intron27	c.3007-1G>C	Splice variant
19	42	ATM ATM CDH	Exon 3 Exon 42 Exon 14	c.94C>T c.6163A>G c.2234A>G	p.Arg32Cys p.Ile2055Val p.Glu745Gly
20	48	MSH6	Exon 4	c.2648A>C	p.Lys883Thr)
21	33	RAD51D	Exon 8	c.775C>T	p.Arg259Trp
22	55	CHEK2	Exon 16￼	c.1690C>T	p.Arg564Trp
23	42	PALB2	Exon 5	c.1699C>T	p.His567Tyr
24	35	RAD50	Exon 19	c.3026A>T	p.Asp1009Val
25	69	ERCC2	Exon 7	c.587G>A	p.Arg196Gln
26	37	MRE11A	Exon 13	c.1480G>A	p.Glu494Lys
27	34	FANCI	Intron 23	c.2456+1G>A	Splice variant

**Supplementary Table S3a.** P/LP variants in the BRCA1 gene.

S. no.	Variant description (HGVS)	Protein change	ClinVar status	Age (years)	Sub type	FH
1	NM_007294.4: c.4508C>A	NP_009225.1: p.Ser1503Ter	P	39	HR	+B
2.	NM_007294.4: c.2820_2830delinsAAGATAAGCCAGTTTGATAA	NP_009225.1: p.Asp940_Cys944delinsGluArgTer	P	48	TNBC	+B
3.	NM_007294.4: c.3627dup	NP_009225.1: p.Glu1210fs	P	50	TNBC	+B
4.	NM_007294.4: c.3770_3771del	NP_009225.1: p.Glu1257fs	P	41	NA	−
5.	NM_007294.4: c.346del	NP_009225.1: p.(Glu116Asnfs*3)	P	42	TNBC	+B
6.	NM_007294.4: c.3607C>T	NP_009225.1: p.(Arg1203Ter)	P	39	TNBC	+
7.	NM_007297.4: c.1935_1939del	NP_009225.1: p.(His692Glnfs*18)	P	49	HR	+
8.	NM_007297.4: c.4520_4521delinsG	NP_009225.1: p.(Pro1554Argfs*5)	P	44	TNBC	+
9.	NM_007297.4: c.4520_4521delinsG	NP_009225.1: p.(Pro1554Argfs*5)	P	46	TNBC	+
10.	NM_007294.4: c.5153-2A>G	Splice acceptor	P	53	TNBC	+
11.	NM_007294.4: c.5053A>G	NP_009225.1: p.(Thr1685Ala)	P	40	TNBC	+
12.	NM_007294.4: c.5509T>C	NP_009225.1: p.(Trp1837Arg)	P	48	HR	+B
13.	NM_007294.4: c.4484+1G>A	Splice donor	P	54	HR	NA
14.	NM_007294.4: c.4065_4068del	NP_009225.1: p.(Asn1355fs)		48	TNBC	+B
15.	NM_007297.4: c.34del	NP_009225.1: p.(Ser59Hisfs*10)	P	34	TNBC	+O
16.	NM_007294.4: c.(?_-232)_(5277+1_5278-1)del	-	P	56	TNBC	−
17.	NM_007294.4: c.68_69del	NP_009225.1: ￼ p.(Glu23fs)	P	47	HR	NA
18.	NM_007294.4: c.1504_1508del	NP_009225.1: p.(Leu502fs)	P	34	TNBC	+
19.	NM_007294.4: c.5152+1G>A	Splice donor	P	35	TNBC	NA
20.	NM_007294.4: c.68_69del	NP_009225.1: p.(Glu23fs)		38	TNBC	NA
21.	NM_007294.4:c.2820_2830delinsAAGATAAGCCAGTTTGATAA	NP_009225.1: p.(Asp940_Cys944delinsGluArgTer)	P	39	TNBC	+O
22.	NM_007294.4:c.5152+5G>A	NP_009225.1:p.?	P	28	TNBC	NA
23.	NM_007294.4: c.4508C>A	NP_009225.1: p.(Ser1503Ter)		31	TNBC	+
24.	NM_007294.4: c.(134+1_135-1)_(441+1_442-1)dup	-	P	64	TNBC	+O
25.	NM_007294.4: c.470_471del	NP_009225.1: p.(Leu156_Ser157insTer)		37	NA	NA
26.	NM_007294.4: c.181T>C	NP_009225.1: p.(Cys61Arg)	P	50	TNBC	−
27.	NM_007294.4: c.5075-1G>C	Splice acceptor	P	26	TNBC	−

+, family history present; −, family history negative; NA, information not available; B, breast cancer in FDR; O, ovarian cancer in FDR; FDR, first degree relative; TNBC, triple negative breast cancer; TP, triple positive; HR, hormone receptor positive

**Supplementary Table S3b.** P/LP variants detected in the BRCA2 gene.

S. no.	Variant description (HGVS)	Protein change	ClinVar status	Age (years)	Sub-type	FH
1	NM_000059.4: c.3182del	NP_000050.3: p. (Lys1061Serfs*16)	P	34	TNBC	+B
2.	NM_000059.4: c.4570_4573del	NP_000050.3: p. (Phe1524fs)	P	55	HR	−
3.	NM_000059.4: c.1909+2T>C	Splice site	LP	33	TNBC	+
4.	NM_000059.4: c.3785C>A	NP_000050.3: p. (Ser1262Ter)	P	43	TP	NA
5.	NM_000059.4: c.126T>A	NP_000050.3: p. (Tyr42*)	P	35	TNBC	+B,O
6.	NM_000059.4: c.347_350del	NP_000050.3: p. (Ser116Ilefs*4)	P	55	TP	+B
7.	NM_000059.4: c.6486_6489del	NP_000050.3: p. (Lys2162fs)	P	36	HR	−

+, family history present; −, family history negative; NA, information not available; B, breast cancer in FDR; O, ovarian cancer in FDR; FDR, first degree relative; TNBC, triple negative breast cancer; TP, triple positive; HR, hormone receptor positive

**Supplementary Table S3c.** P/LP variations in other genes.

S. no.	Gene	Variant description (HGVS)	Protein change	ClinVar status	Age (years)	Sub-type	FH
1	ATM	NM_000051.4: c.5631_5635delinsA	NP_000042.3: p. (Phe1877fs)	P/LP	29	HR	+
2.	TP53	NM_000546.6: c.489C>G	NP_000537.3: p.(Tyr163*)	P	43	TNBC	+B
3.	RAD51D	NM_001142571.2: c.423del	NP_001136043.1): p. (Ala142Glnfs*14)	P	45	TNBC	NA
4.	CHEK2	NM_007194.4: c.409C>T	NP_009125.1: p.(Arg137Ter)	P	45	HR	+B
5.	ATM	NM_000051.4: c.5631_5635delinsA	NP_000042.3: p.(Phe1877fs)	P/LP	40	NA	NA
6.	ATM	NM_000051.4: c.8480T>G	NP_000042.3: p.(Phe2827Cys)	P/LP	65	NA	NA
7.	HMMR	NM_001142556.2: c.1327C>T	NP_001136028.1: p.(Gln443*)	P	34	TNBC	−

+, family history present; −, family history negative; NA, information not available; B, breast cancer in FDR; O, ovarian cancer in FDR; FDR, first degree relative; TNBC, triple negative breast cancer; TP, triple positive; HR, hormone receptor positive
